# Role and Impact of the *brsk2* Gene in Zebrafish Retinal Development and Visual Function Characterized by Behavioral, Histological, and Transcriptomic Analyses

**DOI:** 10.3390/ijms27020858

**Published:** 2026-01-15

**Authors:** Jingxin Deng, Yue Li, Meixin Hu, Chunchun Hu, Jia Lin, Qiang Li, Xiu Xu, Chunxue Liu

**Affiliations:** 1 Division of Child Health Care, Children’s Hospital of Fudan University, National Children’s Medical Center, 399 Wanyuan Road, Shanghai 201102, China; 21111240005@m.fudan.edu.cn (J.D.); llly17849711171@163.com (Y.L.); 22111240011@m.fudan.edu.cn (M.H.); 14211240007@fudan.edu.cn (C.H.); 2 Center for Translational Medicine, Institute of Pediatrics, Shanghai Key Laboratory of Birth Defect, Children’s Hospital of Fudan University, National Children’s Medical Center, 399 Wanyuan Road, Shanghai 201102, China; linjiaalyssa@163.com (J.L.); liq@fudan.edu.cn (Q.L.)

**Keywords:** zebrafish, *BRSK2*, autism spectrum disorder, vision function

## Abstract

Vision is fundamental to the acquisition of motor, cognitive, and social skills, playing a crucial role in typical development. Early visual impairments are associated with various neurodevelopmental conditions, including Autism Spectrum Disorder (ASD). The (Brain-specific serine/threonine-protein kinase 2, *BRSK2*) gene has been identified as a high-risk gene for ASD. This study aims to investigate the role of brsk2 in retinal photoreceptor development and visual function in zebrafish. Using behavioral assays, histological analysis, and transcriptomic profiling, we assessed the impact of *brsk2* deletion on retinal structure and function. The results showed that *brsk2ab*^−/−^ zebrafish larvae exhibited significantly enhanced light perception compared to wild-type (WT) controls. Structural analysis of the retina revealed disruptions in the layered organization, along with up-regulated rhodopsin expression in retinal cells. Additionally, transcriptomic analysis indicated that key opsins and genes involved in visual development and phototransduction pathways were markedly up-regulated following *brsk2* deletion. This research highlights the importance of brsk2 in early retinal circuit development and its potential implications for understanding sensory processing deficits in neurodevelopmental disorders. By linking BRSK2 to specific sensory phenotypes, this study addresses a critical gap in knowledge regarding the mechanisms underlying sensory abnormalities in ASD and related conditions.

## 1. Introduction

Vision serves as the foundation for acquiring motor, cognitive, and social skills and is crucial for typical development. Difficulties in visual perceptual processing affect individuals’ ability to acquire information, receive feedback, and communicate about their physical and social environments [1]. From birth, vision significantly guides motor functions, helps maintain postural control, facilitates grasping and object search, and supports shape recognition [2]. Early facial recognition is critical for building and maintaining interpersonal relationships and for interpreting others’ intentions. Thus, early-onset visual impairments hinder adaptive capability maturation and are intricately linked to various neurodevelopmental conditions [3,4]. Deficits in gaze behavior in infants and young children are considered early core symptoms of Autism Spectrum Disorder (ASD) [5]. Previous studies have shown that over 90% of individuals with ASD exhibited atypical reactions to sensory stimuli [6], particularly abnormal visual perception, which is characterized by either avoiding or seeking additional visual stimuli [7]. Patients with ASD often show heightened sensitivity to local visual details, affecting their overall perception of global visual features [8,9].

BRSK2 protein kinase plays essential roles in neural polarization, axonogenesis and neuronal migration [10,11,12]. Increasing evidence suggests that *BRSK2* is a high-risk ASD gene, with mutations leading to a variety of neurodevelopmental disorder phenotypes including language delay, motor delay and ASD [13,14,15,16,17]. Our previous work established a novel zebrafish model deficient in *brsk2b* and *brsk2ab*, which exhibited prominent ASD-like behaviors [18,19]. Previously reported probands with *BRSK2* mutations exhibited comorbid visual issues, including myopia (p.A158T) [17], hyperopia with eye tremor (p.R65Q) and astigmatism (p.R621C) [13]. This underscores the need for a focused investigation into the specific contributions of BRSK2 to retinal structure and function.

The transparency of zebrafish embryos, along with their suitability for in vitro fertilization and early development, makes them ideal for studying visual function, phototransduction pathway pathology, and molecular mechanisms in animal models [20]. The use of zebrafish allows for the observation of developmental processes in living organisms, facilitating a comprehensive assessment of phenotypic and molecular changes associated with *brsk2* deletion. The human *BRSK2* (NC_000011.10) gene is duplicated into two orthologs, *brsk2a* (NC_007136.7) and *brsk2b* (NC_007118.7) in zebrafish. Notably, the *brsk2a* and *brsk2b* genes in zebrafish are expressed in retina and brain, similar to human beings.

By employing a combination of behavioral assays, histological analysis, and transcriptomic profiling, this research aims to elucidate the effects of *brsk2* deletion on retinal structure and function. Understanding how brsk2 regulates early retinal circuit development is crucial, as impairments in this process may not only contribute to visual dysfunction but also represent a developmental basis for altered sensory processing. Given the strong association of BRSK2 with ASD, this study addresses a significant knowledge gap by linking a key neurodevelopmental gene to specific sensory phenotype, potentially revealing mechanisms underlying sensory abnormalities in neurodevelopmental disorders.

## 2. Results

### 2.1. Enhanced Visual Response in brsk2ab^−/−^ Zebrafish

**Figure 1:**
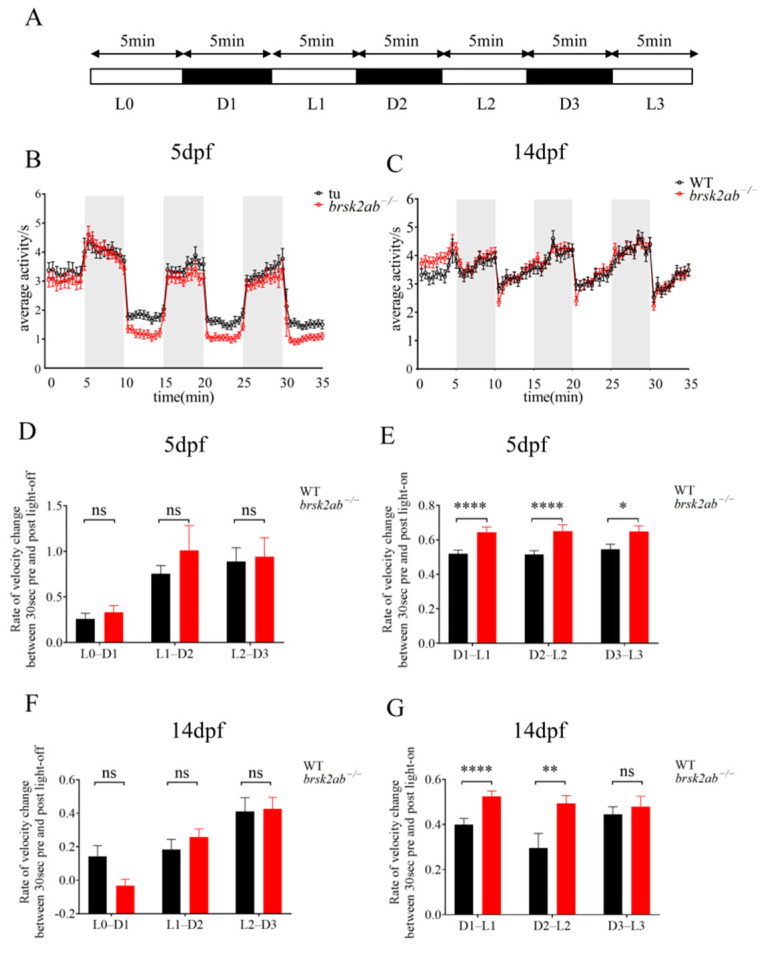
Detection of visuomotor responses in zebrafish. (**A**) Schematic diagram of light/dark test: L0 was the adaptation period, D1, D2, and D3 were dark period, and L1, L2, and L3 were light period. (**B**,**C**) The locomotor activity of the WT and *brsk2ab*^−/−^ larvae at 5 dpf and 14 dpf (**D**,**F**) The rate of velocity change between WT and *brsk2ab*^−/−^ zebrafish at 5 dpf (**D**) and 14 dpf (**F**) during the three 1 min light-dark transitions. (5 dpf: L0–D1 *p* = 0.264; L1–D2: *p* = 0.929; L2–D3: *p* = 0.483; 14 dpf: L0–D1: *p* = 0.08; L1–D2: *p* = 0.324; L2–D3: *p* = 0.721). (**E**,**G**) The rate of velocity change between WT and *brsk2ab*^−/−^ zebrafish at 5 dpf (**D**) and 14 dpf (**F**) during the three 1 min dark-light transitions (5 dpf: D1–L1: *p* < 0.0001; D2–L2: *p* < 0.0001; D3–L3: *p* = 0.013; 14 dpf: D1–L1: *p* < 0.0001; D2–L2: *p* < 0.006; D3–L3: *p* = 0.170). Data are shown as mean ± SEM, 5 dpf: *n* = 48:48, 14 dpf: *n* = 96:96, * *p* < 0.05, ** *p* < 0.01, **** *p* < 0.0001.

To comprehensively assess the visual motion perception ability in *brsk2ab*^−/−^ zebrafish, light/dark tests were performed. As shown in Figure 1, the locomotor activity of *brsk2ab*^−/−^ zebrafish was significantly lower than that of WT zebrafish at 5 days post fertilization (dpf) (Figure 1B) and 14 dpf (Figure 1C). Both genotypes exhibited sensitivity to light changes at 5 dpf and 14 dpf, showing increased activity in the dark and decreased activity in the light (Figure 1B,C). This confirms that phototaxis is an innate behavior of zebrafish larvae in response to light stimulation [20]. We further evaluated the instantaneous change in velocity induced by light alteration to quantify the light sensitivity by comparing the rates of velocity change at the three light-off and three light-on time points between the two genotypes. At 5 dpf and 14 dpf, there was no significant difference in the rate of velocity change between the two genotypes during the 30 s before and after the three light-to-dark transitions (Figure 1D, 5 dpf: L0–D1: *p* = 0.264; L1–D2: *p* = 0.929; L2–D3: *p* = 0.483; Figure 1F, 14 dpf: L0–D1: *p* = 0.08; L1–D2: *p* = 0.324; L2–D3: *p* = 0.721). However, *brsk2ab*^−/−^ zebrafish exhibited a significantly higher rate of velocity change than WT zebrafish during the 30 s before and after transitioning from dark to light (Figure 1E, 5 dpf: D1–L1: *p* < 0.0001; D2–L2: *p* < 0.0001; D3–L3: *p* = 0.013; Figure 1G, 14 dpf: D1–L1: *p* < 0.0001; D2–L2: *p* < 0.006; D3–L3: *p* = 0.170), indicating heightened sensitivity to changes in lighting conditions.

### 2.2. The Body Color of brsk2ab^−/−^ Zebrafish Exhibits a Darkened Pigmentation

**Figure 2:**
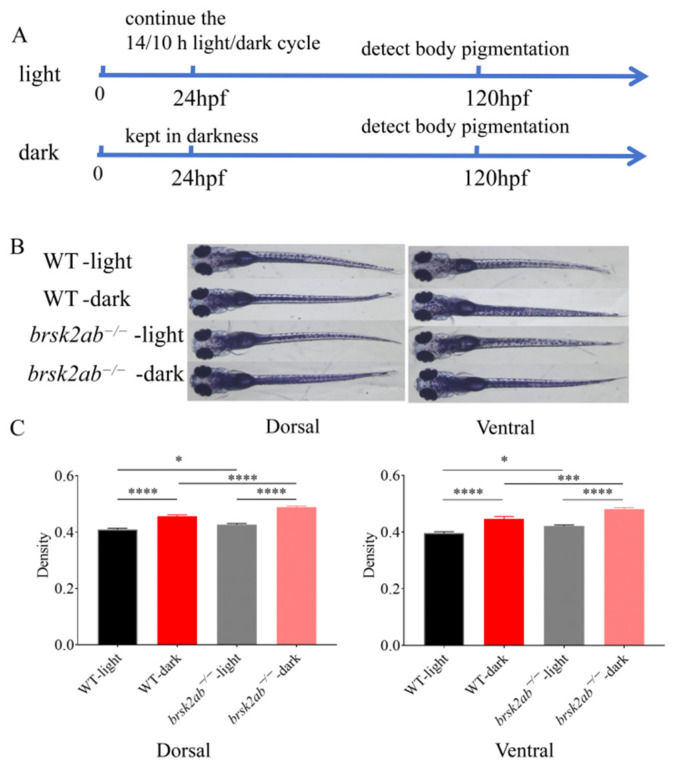
Visually induced analysis of body color in zebrafish. (**A**) Schematic representation of the experimental design. (**B**) Gross morphology of the ventral and dorsal surfaces of WT and *brsk2ab*^−/−^ zebrafish at 5 dpf. (**C**) Statistical analysis of the average optical density of pigmentation on the ventral and dorsal surfaces of the zebrafish. Data shown as mean ± SEM, *n* = 15:15 * *p* < 0.05, *** *p* < 0.001, **** *p* < 0.0001.

The increased rate of velocity changes observed in *brsk2ab*^−/−^ zebrafish larvae in response to light cannot be solely attributed to their motor function, given their reduced spontaneous locomotion. Therefore, we hypothesized that *brsk2* deletion might affect the visual function of zebrafish. To investigate this, we analyzed the body color pigmentation in zebrafish. Zebrafish can adjust their body coloration in response to environmental light changes by regulating melanin dispersion or accumulation in melanocytes, which aids in camouflage and predator avoidance. This mechanism reflects processes in the retina, pineal gland, deep brain, and melanocytes [21]. WT and *brsk2ab*^−/−^ zebrafish embryos were subjected to light-sheltered conditions starting at 24 h post fertilization (hpf) and assessed for body melanin pigmentation at 5 dpf (Figure 2A). The findings indicated that both WT and *brsk2ab*^−/−^ zebrafish exhibited sensitivity to light-induced changes in melanin levels. The *brsk2ab*^−/−^ zebrafish displayed darker body pigmentation compared to WT zebrafish under normal lighting conditions (Figure 2C, dorsal side: *p* = 0.0465; ventral surface: *p* = 0.022). A similar pattern was observed in light-sheltered zebrafish (Figure 2C, dorsal surface: *p* < 0.0001; ventral surface: *p* = 0.0007), suggesting that *brsk2ab*^−/−^ zebrafish are more responsive to changes in light exposure

### 2.3. The Retinal Structure Was Found to Be Altered in brsk2ab^−/−^ Larvae

**Figure 3:**
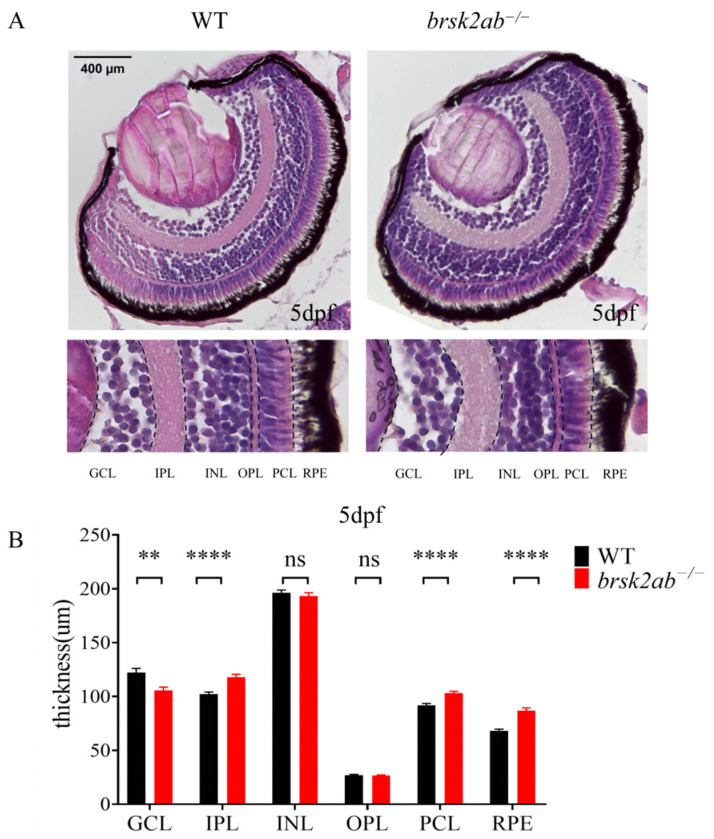
HE staining of zebrafish retinas at 5 dpf. (**A**) GCL, ganglion cell layer; IPL, inner plexiform layer; INL, inner nuclear layer; OPL, outer plexiform layer; PCL, photoreceptor layer; RPE, pigment epithelial layer. Scale bar 400 μm. (**B**) Statistical analysis of thickness of each retinal cell layer. (GCL: *n* = 50:45, *p* = 0.0014; IPL: *n* = 51:53, *p* < 0.0001; INL: *n* = 51:53, *p* = 0.446; OPL: *n* = 51:53, *p* = 0.739; PCL: *n* = 51:53, *p* < 0.0001; RPE: *n* = 51:52, *p* < 0.0001). Data shown as mean ± SEM, ** *p* < 0.01, **** *p* < 0.0001.

Since photoreceptor function was altered in *brsk2ab*^−/−^ zebrafish, we first assessed whether this was due to abnormal structural development of the retina. We examined retinal structure and morphology in zebrafish eyes using paraffin sections and HE staining. As shown in Figure 3, the ganglion cell layer (GCL) of *brsk2ab*^−/−^ zebrafish at 24 hpf exhibited a reduction in thickness (*p* = 0.0014). Conversely, the inner plexiform layer (IPL) (*p* < 0.0001), the photoreceptor layer (PCL) (*p* < 0.0001) and the retinal pigment epithelium (RPE) (*p* < 0.0001) were observed to be thickened. However, no significant difference was found in the inner nuclear layer (INL) (*p* = 0.446) and outer plexiform layer (OPL) (*p* = 0.739). This indicates that the loss of brsk2 impacts the development of all retinal cell layers at early developmental stages.

**Figure 4:**
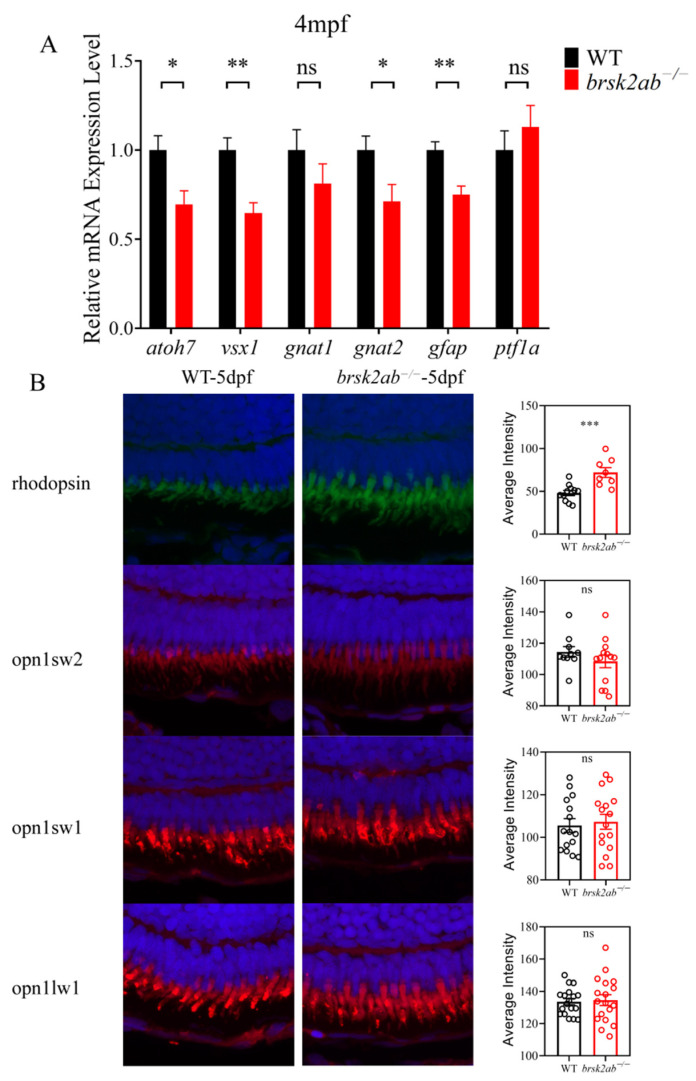
Detection of each cell layer and photoreceptor markers in the retina of *brsk2ab*^−/−^ zebrafish. (**A**) mRNA levels of markers in each cell layer of zebrafish retina after *brsk2* deletion. *atoh7* (*p* = 0.033), vsx1 (*p* = 0.008), *gnat2* (*p* = 0.011), *gfap* (*p* = 0.009), *gnat1* (*p* = 0.28) and *ptf1a* (*p* = 0.461). (*n* = 4:4) (**B**) The fluorescence intensity of retinal opsins of zebrafish at 5 dpf (rhodopsin: *n* = 11:8, *p* = 0.009; opn1sw2: *n* = 10:13, *p* = 0.291; opn1sw1: *n* = 15:16, *p* = 0.7151; opn1lw1: *n* = 18:18, *p* = 0.798). Data shown as mean ± SEM, * *p* < 0.05, ** *p* < 0.01, *** *p* < 0.001, ns *p* > 0.05.

To identify the affected cell types in the *brsk2ab*^−/−^ zebrafish retina, we examined markers for specific cell subclasses. As shown in Figure 4A, the results revealed significantly reduced relative mRNA expression levels of *atoh7* (a marker of GCL, *p* = 0.033), *vsx1* (a marker of bipolar cells, *p* = 0.008), *gnat2* (a marker of cone photoreceptors, *p* = 0.011), and *gfap* (a marker of astrocytes, *p* = 0.009) in *brsk2ab*^−/−^ zebrafish at 4 months post fertilization (mpf). In contrast, no significant difference was observed in the expression levels of *gnat1* (a marker of rod photoreceptors, *p* = 0.28) and *ptf1a* (a marker of retinal progenitor cells, *p* = 0.461). Cones and rods are two types of photoreceptor cells in the PCL of the retina, each with distinct roles in light perception. To investigate potential differences in photoreceptor populations, we used specific antibodies (rhodopsin, opn1sw2, opn1sw1 and opn1lw1) to label rods and cones (blue, UV and red cones) in retinal sections at 5 dpf. Figure 4B shows no differences among the various cone subtypes (blue cones: *p* = 0.291; UV: *p* = 0.7151; red: *p* = 0.798). However, in *brsk2ab*^−/−^ zebrafish, the rhodopsin fluorescence intensity in the retina significantly increased (*p* = 0.009).

### 2.4. Changes in Photoconduction Pathways in brsk2ab^−/−^ Zebrafish

**Figure 5:**
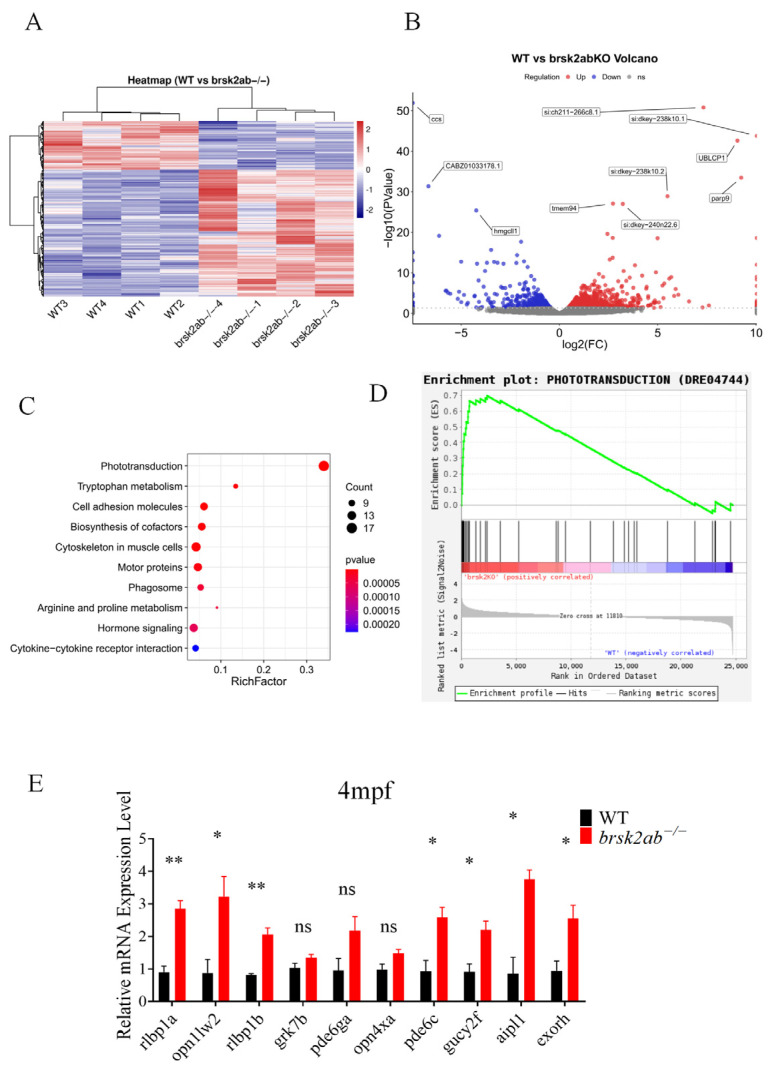
Transcriptomic analysis revealed aberrant activation of the phototransduction pathway in *brsk2ab*^−/−^ zebrafish. Heat map (**A**) and volcano plot (**B**) for differential gene expression between WT and *brsk2ab*^−/−^ zebrafish. (**C**) KEGG pathway enrichment analysis of the genes identified to be differentially expressed between WT and *brsk2ab*^−/−^ groups. The significantly enriched pathways are shown at the phototransduction pathway. (**D**) GSEA of genes in the phototransduction pathway (DRE04744). (**E**) Relative expression of genes involved in the phototransduction pathway was detected by qRT-PCR. (*n* = 4) Data shown as mean ± SEM, * *p* < 0.05, ** *p* < 0.01, ns *p* > 0.05.

To understand how the loss of *brsk2* affects photoreceptor function, we performed a transcriptomic analysis using brain tissues from WT and *brsk2ab*^−/−^ zebrafish at 4 mpf. The analysis revealed significant differential expression of 906 genes between the two groups, with 655 genes up-regulated and 251 genes down-regulated (Figure 5A,B). Additionally, KEGG pathway analysis indicated that differentially expressed genes were primarily involved in phototransduction, tryptophan metabolism, and cell adhesion molecules (Figure 5C). Gene Set Enrichment Analysis (GSEA) also revealed a strong correlation between phototransduction and the *brsk2ab*^−/−^ zebrafish gene sets (Figure 5D). Key genes in the phototransduction pathway identified by KEGG analysis were validated at the mRNA level using zebrafish eye tissues at 4 mpf. The results showed significant up-regulation in the relative expression levels of *rlbp1a* (*p* = 0.0031), *rlbp1b* (*p* = 0.0035), *opn1lw2* (*p* = 0.0346), *pde6c* (*p* = 0.0227), *gucy2f* (*p* = 0.0236), *aipl1* (*p* = 0.0282) and *exorh* (*p* = 0.0325). However, no differences were observed in the relative expression levels of *grk7b* (*p* = 0.154), *pde6ga* (*p* = 0.095), *opn4xa* (*p* = 0.074) (Figure 5E). These findings indicate that the photoconduction pathway was disrupted in *brsk2ab*^−/−^ zebrafish.

## 3. Discussion

The significance of vision in the development of motor, cognitive, and social skills is well-established, as visual perceptual processing is essential for effective information acquisition and interaction with one’s environment. Current research indicates that visual impairments from an early age can severely hinder adaptive capabilities, particularly in the context of neurodevelopmental disorders such as ASD. Notably, deficits in gaze behavior are recognized as early indicators of ASD, with a substantial proportion of affected individuals exhibiting atypical sensory responses, especially in visual perception. The role of BRSK2, a protein kinase involved in neural development, has emerged as a critical factor in understanding these sensory processing abnormalities. Previous findings have linked *BRSK2* mutations to various visual impairments, underscoring the need for targeted investigations into its influence on retinal structure and function.

This study employs a well-established zebrafish model, allowing for a comprehensive analysis of behavioral and molecular changes associated with *brsk2* loss. Key findings indicate that *brsk2ab*^−/−^ zebrafish exhibited altered locomotor activity in response to light changes, structural modifications in the retina, and disruptions in phototransduction pathways, suggesting brsk2′s role in normal zebrafish visual development and function.

Initially, we observed a significant increase in the transition rate from darkness to light during early development in *brsk2ab*^−/−^ zebrafish. By 5 dpf, these larvae exhibited a darker body color than WT zebrafish. Zebrafish display vision-guided behaviors, and respond with a series of locomotor actions to changes in ambient illumination [20,22]. In addition, larval zebrafish may regulate melanin distribution in response to ambient light changes, a process influenced by ocular photoreception [21]. Our data revealed enhanced sensitivity to light stimuli in *brsk2ab*^−/−^ zebrafish. The process of visual function involves phototransduction in photoreceptors, synaptic communication, and neuromodulation [23]. During phototransduction, the initial light signals captured by retinal rods and cones are transduced within the retina, converting light energy into neural signals. These signals are then transmitted via the optic nerve to the lateral geniculate nucleus in the thalamus. Calcium-dependent phototransduction is the primary mechanism of light adaptation in vertebrate photoreceptors, as widely recognized in previous research [24]. Although the role of brsk2 in visual adaptation is not well understood, previous studies confirm that brsk2 kinase activation is influenced by [cAMP]/[Ca2+]-dependent signaling pathways, which regulate glucose-stimulated insulin secretion in pancreatic β-cells [24,25]. We infer that brsk2 may modulate the response to light stimuli through calcium signaling pathways in zebrafish.

Secondly, this study observed significant changes in the retinal layered structure of *brsk2ab*^−/−^ larvae. The RPE absorbs stray light, forms the blood-retina barrier and regenerates visual pigments [26]. Photoreceptors (rods and cones) reside in the PCL, extending their inner and outer segments toward the adjacent RPE. These specialized cells detect photons and transmit information via chemical signaling [27]. The cell bodies of bipolar cells, horizontal cells, and amacrine cells are located in INL, which lies between the photoreceptors and ganglion cells. Their primary function is to extract luminance contrast, color contrast, and movement features from visual information processed by the RPE. The IPL and OPL contain synaptic connections among these retinal neurons. The GCL, situated in the innermost part of the retina, houses the cell bodies of retinal ganglion cells (RGCs), the output neurons of the retina. These RGCs extend their axons through the optic nerve and tract to reach specific brain regions [28]. Given the retina’s crucial functions, abnormalities in these layers may impair the functioning of visual processing centers. The thickening of the RPE, PCL, IPL layers suggests that the procedures of light absorbing, photon detection, and optical information processing in *brsk2ab*^−/−^ larvae could be enhanced. In contrast, the transmission of processed visual information via the optic nerve may be impaired, as the GCL layer was found to be thinner. Based on these data alone, it is difficult to determine whether the visual function of *brsk2ab*^−/−^ zebrafish is altered positively or negatively. Therefore, the mRNA expression levels of specific cell layer markers were examined. The results showed that the expression of *atoh7*, *vsx1*, *gnat2*, and *gfap* was significantly decreased in *brsk2ab*^−/−^ zebrafish. This indicates that the optic signal conduction processes mediated by the GCL and opsins in the PCL layer could be disrupted following *brsk2* deficiency. Previous studies have revealed that BRSK2 plays a role in neuronal axon formation in brain neurons, as neurons from *Brsk2* knockout mice exhibit a loss of normal polarity. This process may be mediated by BRSK2′s phosphorylation of microtubule-associated proteins, which in turn affects cytoskeletal organization. Therefore, we can speculate that BRSK2 may influence the axonogenesis of retinal ganglion cells through a similar mechanism [10,11,12]. Further analysis of opsins suggests that rod cells might be the most affected subclass, with potential impact on light adaptation. Opsins are light-sensitive proteins with unique sensitivities to different wavelengths, located in the outer segments of rod and cone photoreceptors [29,30]. Opsins change structure upon absorbing photons, triggering electrical signals in the PCL. These visual signals are then transmitted through retinal cells and eventually sent to the visual center of the cerebrum via ganglia cells [31]. Increasing evidence suggests that retinoid cycle dysfunction causes vision defects such as macular degeneration and retinitis pigmentosa [32]. In the dark, all-trans retinal is converted to 11-cis retinal, which binds to opsin to form rhodopsin—a process essential for dark vision. Conversely, in light, the two separate and rhodopsin is broken down, a process known as the retinoid cycle. Here we detected an accumulation of rhodopsin in *brsk2ab*^−/−^ larvae, which may be associated with alteration in the phototransduction cascade, subsequently affecting visual motor responses. However, it remains unclear whether this abnormal accumulation results from increased conversion of all-trans retinal to 11-cis retinal or impaired rhodopsin breakdown in response to light. Further investigation is needed to elucidate the underlying molecular and cellular mechanisms. Additionally, *Brsk2* deficiency potentially disrupt neuronal polarization by interfering with the pruning of neuronal protrusions in mice [33]. In pancreatic β-cells, genetic ablation of BRSK2 enhances glucose-stimulated insulin secretion and leads to a concomitant reduction in blood glucose levels [34]. Collectively, the available evidence suggests a potential inhibitory function for BRSK2, although direct experimental validation remains to be established.

Although significant progress has been made in recent years in understanding light signal transduction, the roles of specific genes in photoreceptors, particularly the *brsk2* gene, remain poorly characterized. Therefore, we investigated whether defects in brsk2 disrupt normal brain function and affect the responsiveness to visual stimuli. This study integrated transcriptomic analysis and GSEA to comprehensively assess the impact of *brsk2* deletion on photoreceptor function. In the transcriptomic analysis, we focused on expression changes of genes involved in the visual transduction signaling pathway and their associated genes. The results revealed a significant up-regulation of genes in the phototransduction pathway in *brsk2ab*^−/−^ zebrafish, suggesting hyperactivity in phototransduction. This finding is consistent with the behavioral and morphological phenotypes observed in the eyes. Visual function integrity involves not only the reception of diverse visual signals by the retina and their transmission through the optic nerve but also the processing and feedback of these signals by the visual center in the cerebral cortex [35]. Neurological lesions can also lead to abnormal visual experiences, and visual impairment resulting from neurodevelopmental disorders rather than ocular structural defects is referred to as cerebral visual impairment [35]. The primary visual anomaly in individuals with ASD often involves disrupted processing of sensory cues, leading to atypical visual perception [7]. Recent studies suggest that the link between ASD and cerebral visual impairment may originate from developmental abnormalities in early brain regions involved in social and adaptive functions, including the neural network responsible for visual perception [4]. Further analysis of gene functions and cellular behavior revealed that the deletion of *brsk2* significantly impairs the function of photoreceptor cells, consequently affecting visual performance. Specifically, the expression of several key genes directly or indirectly involved in the visual transduction pathway, such as rlbp1a and rlbp1b, was up-regulated, indicating disruption of the visual transduction pathway. This phenomenon highlights the adaptive mechanisms of photoreceptors under varying environmental conditions and offers new insights into understanding complex biological systems. The complexity and diversity of the light signal transduction process imply that research on specific genes remains both challenging and promising.

Indeed, there is no literature examining retinal structural changes and abnormal expression of rhodopsin in the *brsk2ab*^−/−^ zebrafish model. The relationship between BRSK2 and autism has only been clarified in recent years, and many aspects still need further research and exploration. Abnormalities in retinal structure have already been observed in individuals with ASD compared to typical development (TD) individuals [36,37,38,39]. Similar structural and functional abnormalities in the visual cortex and retina have also been observed in ASD mouse models such as BTBR and Fmr1, En2 knockout mice [40,41,42]. Particularly, previous research has found a significant reduction in the thickness of the retinal GCL in ASD mice, suggesting damage to retinal neurons, which is consistent with the results of this study [42].

Research limitations are evident in this investigation. First, the assessment of visual function via the light/dark test may be confounded by the zebrafish’s motor performance. The reliance on a zebrafish model, despite its advantages, may not fully recapitulate the complexity of human visual processing. Second, the study’s focus on behavioral and molecular outcomes may overlook other potential neurodevelopmental factors influenced by *BRSK2*, thus providing a limited perspective on the gene’s broader implications in visual perception and sensory integration. Third, the limited number of visual transduction genes showing significant changes may result from methodological factors, such as the sensitivity of gene selection and temporal discrepancies between RNA-seq and qRT-PCR experiments. Finally, the absence of clinical validation further underscores the need to establish the relevance of these findings to human health, necessitating future studies aimed at bridging these gaps. Addressing these constraints in future research will be essential for a more comprehensive understanding of BRSK2′s role in neurodevelopmental disorders.

## 4. Materials and Methods

### 4.1. Zebrafish Maintenance and Husbandry

The maintenance protocols for the Tu strain zebrafish were detailed in previous studies [19]. The fish were housed in a standard recirculation system under a 14–10 h light-dark cycle for at least three months before breeding. The system was supplied with reverse osmosis-filtered water (pH 7.0–7.5), and the temperature was maintained at 28 °C. All zebrafish experiments were conducted at the Center for Translational Medicine, Children’s Hospital of Fudan University, following approval from the institutional animal care committee and in compliance with both local and international regulations.

### 4.2. Generation of brsk2 Mutants by CRISPR/Cas9

**Figure 6:**
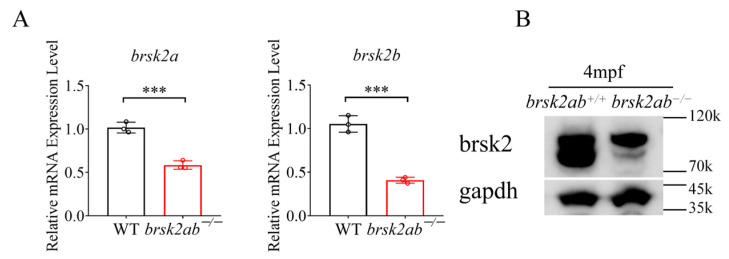
(**A**) RT-qPCR showed that the mRNA expression levels of *brsk2a* and *brsk2b* were decreased in *brsk2ab*^−/−^ zebrafish. (**B**) Western blot showed that the expression of brsk2 protein was significantly reduced in *brsk2ab*^−/−^ zebrafish. *** *p* < 0.001.

The *brsk2* mutants were generated using the CRISPR/Cas9 system as previously described [19]. The target sequences for *brsk2a* and *brsk2b* were 5′–GGAGCAGCCGGTACCCAGGA–3′ and 5′–GGGCAGGTTAACACCCAAAG–3′, respectively. After raising the embryos for 24 h, genomic DNA was extracted to assess mutagenic efficiency using the primers listed in Table 1 and Sanger sequencing. Subsequently, the mRNA and protein expression levels in the *brsk2ab* double mutants obtained by crossing *brsk2a* and *brsk2b* mutants, were measured to validate the model (Figure 6).

### 4.3. Light/Dark Behavioral Test

The light/dark test paradigm, used to assess phototaxis, was described in previous studies [18,43]. The activity of zebrafish larvae at 5 dpf and 14 dpf was measured using the Viewpoint system and the video track module of Zebralab software (https://www.viewpoint.com, accessed on 31 December 2025). Larvae were placed in a 24-well plate, and the experiment was conducted at a constant temperature of 28 °C for 35 min. This duration included a 5 min adaptation period (L0) and three dark/light cycles. Each cycle consisted of 5 min of darkness followed by 5 min of light, denoted as D1, L1, D2, L2, D3, and L3 in Figure 1A. To detect changes in activity under intermittent light stimuli, the light intensity was set at 100 lx during the light periods and 0 lx during the dark periods. The detection threshold for the activity trajectory was set to 25, with a video frame rate of 25/s. The analysis included the average distance moved per 30 s interval, and activity changes during the light-to-dark and dark-to-light transitions for each larva. The formulas used to calculate velocity changes 30 s before and after the lights were turned on or off were as follows:

(1) $$\mathrm{rate}\;\mathrm{of}\;\mathrm{velocity}\;\mathrm{change}\;\mathrm{of}\;\mathrm{light}\;\mathrm{to}\;\mathrm{dark}\;\mathrm{transition}=\frac{\mathrm{velocity}\;\mathrm{for}\;30\;s\;\mathrm{after}\;\mathrm{light}\;\mathrm{off}-\mathrm{velocity}\;\mathrm{for}\;30\;s\;\mathrm{before}\;\mathrm{light}\;\mathrm{off}}{\mathrm{velocity}\;\mathrm{for}\;30\;s\;\mathrm{before}\;\mathrm{light}\;\mathrm{off}}$$

(2) $$\mathrm{rate}\;\mathrm{of}\;\mathrm{velocity}\;\mathrm{change}\;\mathrm{of}\;\mathrm{dark}\;\mathrm{to}\;\mathrm{light}\;\mathrm{transition}=\frac{\mathrm{velocity}\;\mathrm{for}\;30\;s\;\mathrm{before}\;\mathrm{light}\;\mathrm{on}-\mathrm{velocity}\;\mathrm{for}\;30\;s\;\mathrm{after}\;\mathrm{light}\;\mathrm{on}}{\mathrm{velocity}\;\mathrm{for}\;30\;s\;\mathrm{before}\;\mathrm{light}\;\mathrm{on}}$$

### 4.4. Body Color Detection

Zebrafish embryos are initially transparent but begin to develop pigmentation by 24 hpf. Zebrafish pigmentation is influenced by light, and light-induced pigment aggregation becomes dominant by 120 hpf [21,44]. WT and *brsk2ab*^−/−^ zebrafish were maintained under a standard 14/10 h light/dark cycle until 120 hpf as control groups (referred to as “light” in Figure 2A), while others were kept in darkness for 4 days starting at 24 hpf as experimental groups (referred to as “dark” in Figure 2A). At 120 hpf, body pigmentation on both the dorsal and ventral sides of larvae from control groups under normal light conditions and experimental groups after dark treatment was observed using a stereo microscope (Leica M205 FA). The mean melanin density was quantified with Fiji (ImageJ) software (https://imagej.net/ij/, accessed on 31 December 2025). and expressed as InDen/area. Twenty-four larvae were randomly selected and measured per group, with three replicates conducted for each group.

### 4.5. Assessment of Histological Changes

At 5 dpf, larvae were fixed in 4% paraformaldehyde overnight at 4 °C. After fixation, the larvae were dehydrated through a series of ascending ethanol concentrations (50%, 70%, 80%, 95%, and 100%), with each step lasting for 1 h. The tissues were then embedded in paraffin and sectioned into 5 μm-thick slices. From each eyeball tissue, 2 to 8 consecutive slices from the region with the largest diameter were selected and stained with hematoxylin and eosin (H&E) according to the manufacturer’s instructions (solarbio, G1120). The thickness of the retinal cell layers was analyzed using ImageJ software, with the maximum diameter of the peripheral retina in each slice selected for measurement and comparison.

### 4.6. Immunofluorescence in Zebrafish

Larvae were fixed at 5 dpf, embedded in paraffin, and sectioned into 5 μm thick slices. The sections were deparaffinized in xylene for 10 min followed by immersion in a descending alcohol series (100%, 95%, 80%) for 5 min each. The tissue sections were then treated with 3% H_2_O_2_ at room temperature for 5 min, washed with distilled water, and rinsed with phosphate-buffered saline with tween (PBST) for 5 min each. Antigen retrieval was performed using citrate buffer heated in a microwave on medium power for 8 min, cooled for 8 min, and then heated again on medium-low power for 7 min. After three 5 min washes with phosphate-buffered saline (PBS), the samples were blocked with Immunol Staining Blocking Buffer (Beyotime, Shanghai, China, P0102) for 30 min. The primary antibodies, diluted at a ratio of 1:200 in blocking buffer, were incubated with the samples overnight at 4 °C. Subsequently, the samples were washed three times with PBS before being incubated with secondary antibodies (diluted at a ratio of 1:200 in blocking buffer) in the dark for one hour at room temperature. Finally, the zebrafish specimens were washed three times with PBS and visualized under a confocal microscope (Leica M205FA, Wetzlar, Germany). For the confocal imaging of each sample in both groups, the microscope parameters were kept consistent, including laser intensity, field of view, resolution setting (1024 × 1024), Z-step size and number, smart gain, smart offset, etc. The fluorescence images of each sample were analyzed using ImageJ software to detect and compare the average intensity.

The following antibodies were used: Anti-Rhodopsin (Abcam, Waltham, MA, USA, ab183399), Anti-Opn1sw1 (customized), Anti-Opn1lw1 (customized), and Anti-Opn1sw2 (Abgent, San Diego, CA, USA, Azb21565b), Alexa Fluor 488-affinipure Goat anti-mouse IgG (H+L) (Jackson lab, Bar Harbor, ME, USA, 115-545-003), Alexa Fluor 594 Donkey anti-rabbit (Abcam, A21207).

### 4.7. Western Blotting

Zebrafish eye tissues were dissected and lysed in cold Radio Immunoprecipitation Assay (RIPA) lysis buffer (Beyotime, P0013B) supplemented with protease inhibitor cocktail (Beyotime, P1045) at a 100:1 ratio for 30 min on ice. The lysate was then centrifuged at 12,000 rpm for 15 min at 4 °C to collect the supernatant, and the protein concentration was determined using a bicinchoninic acid (BCA) protein assay kit (Beyotime, P0014). Equal amounts of protein samples were separated by 10% SDS-PAGE and transferred to polyvinylidene fluoride (PVDF) membranes (Millipore, Burlington, MA, USA, IPVH00010). After blocking with skim milk in tris-buffered saline with tween20 (TBST) for 1 h at room temperature, the membranes were incubated overnight at 4 °C with anti-brsk2 (1:500, custom) and anti-GAPDH (1:5000, Proteintech, Rosemont, IL, USA, 60004-1-Ig) antibodies. The next day, the membranes were washed three times with TBST for 10 min each and then incubated with horseradish peroxidase-conjugated IgG secondary antibody (1:5000, Affinity, Tokyo, Japan, #S0001) at room temperature for 1 h. After another three washes with TBST for 10 min each, protein bands were visualized using an electrochemiluminescence (ECL) kit (biosharp, Hefei, China, BL520A). All experiments were performed in triplicate and protein expression levels were normalized to β-actin.

### 4.8. Quantitative Real-Time Polymerase Chain Reaction (qRT-PCR)

Zebrafish eye tissues were collected at 4 mpf and used for qRT-PCR analysis. Total RNA was extracted using the RNAiso plus kit (No. 9108, Takara Biomedical Technology, Beijing, China) according to the manufacturer’s protocol. Reverse transcription was performed using the PrimeScript™ RT reagent Kit (RR047, Takara Biomedical Technology) according to the instructions. The resulting cDNA was used in a qRT-PCR system with TB Green Premix Ex Taq II (Tli RNaseH Plus) (RR820A, Takara Biomedical Technology) on a QuantStudio Real-Time PCR Systems (Thermo Fisher, Waltham, MA, USA). Relative mRNA levels normalized to β-actin were calculated using the 2^−∆∆Ct^ method. Each group consisted of four samples, with three technical replicates per sample, and the experiment was performed with three biological replicates. GraphPad Prism 8.0 software was used for bar charts and performing statistical analysis. Primer sequences for qRT-PCR are listed in Table 1.

### 4.9. RNA-Seq and Bioinformatics Analysis

Total RNA was extracted from zebrafish brain tissue at 4 mpf using Trizol Reagent (Invitrogen Life Technologies, Carlsbad, CA, USA, 15596018CN) between 4 and 5 p.m. RNA concentration, quality and integrity were assessed using a NanoDrop spectrophotometer (Thermo Scientific, USA). The sequencing library was subsequently sequenced on the NovaSeq 6000 platform (Illumina, San Diego, CA, USA) by Suzhou PANOMIX Biomedical Tech Co., Ltd. (Suzhou, China). The reference genome and gene annotation files were obtained from a genome database. Filtered reads were aligned to the reference genome (zebrafish GRCz11) using HISAT2 v2.0.5.3 software. HTSeq (0.9.1) was used to calculate read counts for each gene as the raw expression level, while FPKM was used to standardize expression levels. Differential gene expression analysis was performed using DESeq with the following thresholds: |log2FoldChange| > 1 and significance level of *p* < 0.05. KEGG pathway enrichment analysis of differentially expressed genes was performed using Cluster Profiler (3.4.4) software, focusing on significantly enriched pathways (*p* < 0.05). GSEA and volcano plot analysis were performed using the ggplots2 package in R software (https://www.r-project.org/, accessed on 31 December 2025).

### 4.10. Statistical Analysis

GraphPad Prism 8.0 software was used for statistical analysis. Student’s *t*-test was used to compare behavioral test results, retinal cell layers thickness, fluorescence intensity, and mRNA expression levels between WT and *brsk2ab*^−/−^ zebrafish. One-way ANOVA test was used for body color analysis. Data are expressed as mean ± SEM, * *p* < 0.05, ** *p* < 0.01, *** *p* < 0.001, **** *p* < 0.0001.

## 5. Conclusions

In conclusion, the research demonstrates that brsk2 plays a pivotal role in retinal development and function, particularly in visual motion sensing. Our findings revealed enhanced light responsiveness, altered retinal structure, and abnormal activation of the phototransduction pathway in the central nervous system of *brsk2ab*^−/−^ zebrafish. These results provide insight into visual processing deficits associated with neurodevelopmental disorders such as ASD. The findings underscore the importance of BRSK2 in influencing sensory circuit development, potentially revealing mechanisms underlying sensory abnormalities. This study significantly enhances understanding of how genetic factors contribute to visual perceptual challenges in neurodevelopmental conditions, paving the way for future therapeutic strategies.

## Figures and Tables

**Table 1:** Primers for PCR genotyping and qRT-PCR probes used in this study.

Item	Oligo Name	Sequence (5′–3′)	Length (bp)
*brsk2a* genotyping	*brsk2a*-gRNA-I9-PCR-F	GAAATAAATGTAGGTTGGGACTAGTGG	345
	*brsk2a*-gRNA-I10-PCR-R	CACACTCCAAACATTAGCCATTTAC	
*brsk2b* genotyping	*brsk2b*-gRNA-I3-PCR-F	GCAGTAGTTCTACCATGGGATTTC	236
	*brsk2b*-gRNA-I4-PCR-R	AGGCTTCTGACTCACTGAGATTTG	
*brsk2a*	*brsk2a*-E1-qPCR-F	AGCACTACCCCTCATGCCAA	84
	*brsk2a*-E1/2-qPCR-R	AAGCTTTACAAGACCTGTCTGTCCT	
*brsk2b*	*brsk2b*-E12-qPCR-F3	TCTCCACTTTTGACGAGGCA	131
	*brsk2b*-E12/13-qPCR-F3	TTACTCTGCCCGTGTTGTGC	
*β-actin*	*β-actin*-E2-qPCR-F	CGAGCTGTCTTCCCATCCA	102
	*β-actin*-E3-qPCR-R	TCACCAACGTAGCTGTCTTTCTG	
*ribp1a*	*ribp1a*-qPCR-F	AGCCGGTTACCCACGAATCC	118
	*ribp1a*-qPCR-R	GTAGGCACGCAGGGTCTCATC	
*gucy2f*	*gucy2f*-qPCR-F	TGCTGGATCTCATCAAGGGCA	131
	*gucy2f* -qPCR-R	CAGGCCGAGCATGCAATACG	
*opn1lw2*	*opn1lw2*-qPCR-F	ATGAACCGACAGTTCCGCGT	253
	*opn1lw2*-qPCR-R	CAGTTTGAGTCCAGCATTGCCA	
*ribp1b*	*ribp1b*-qPCR-F	CCATTGAGGCCGGATACCCA	285
	*ribp1b* -qPCR-R	GGGCAGGGAAAGAGTCCTGC	
*grk7b*	*grk7b*-qPCR-F	TGGGCTTTGGGCTGTAGCAT	220
	*grk7b*-qPCR-R	GCTTCCGAGGGTCATCATTGC	
*pde6ga*	*pde6ga*-qPCR-F	AAAGGGTGTCATCGGATTTGGTG	97
	*pde6ga*-qPCR-R	CAGGTGACTGTACGCCTCCC	
*aipl1*	*aipl1*-qPCR-F	ATCAACCAGCACCCTGGGAC	244
	*aipl1*-qPCR-R	CTAGGGAGTCCTGCTCGCTC	
*opn4xa*	*opn4xa*-qPCR-F	ATGTGACGTCCACTCCTGCC	141
	*opn4xa*-qPCR-R	GCTTCTCCACATCCCTGCTTG	
*pde6c*	*pde6c*-qPCR-F	TGGAGCCGTATAAAGGCCCG	119
	*pde6c*-qPCR-R	GTCTGCAGGAGGGTTCGGTA	
*exorh*	*exorh*-qPCR-F	ATCATGCTCAACAGACAGTTCCG	142
	*exorh*-qPCR-R	CTGGAGACACCTGAGCGGAG	
*atoh7*	*atoh7*-qPCR-F	CCTGAACACGGCATTCGACC	132
	*atoh7*-qPCR-R	GCGTCGCTCAGGATCCGATT	
*vsx1*	*vsx1*-qPCR-F	GCTCCCTGGCTTCTAGGTGA	258
	*vsx1*-qPCR-R	GCTGTTTTCTGTGCGAGGCG	
*gnat1*	*gnat1*-qPCR-F	AGGATTCAGGCATCCAGGCG	103
	*gnat1*-qPCR-R	CCGGGCTGGATCAACCTCTC	
*gnat2*	*gnat2*-qPCR-F	CGACGAGATGGGTAGCGGAGC	268
	*gnat2*-qPCR-R	GCCCCTGATGATGGCCAGAGC	
*gfap*	*gfap*-qPCR-F	ACTGAGGAGTGGTATCGCTCAAA	150
	*gfap*-qPCR-R	AGACCCACGGAGAGATTCCA	
*ptf1a*	*ptf1a*-qPCR-F	CCCAGACTACGGGTTGCCTC	102
	*ptf1a*-qPCR-R	GTCCAGACTTTCGCTGTCCG	

## Data Availability

The original contributions presented in this study are included in the article. Further inquiries can be directed to the corresponding author.
